# Helicobacter pylori infection increases the risk of nonalcoholic fatty liver disease: Possible relationship from an updated meta-analysis

**DOI:** 10.1097/MD.0000000000034605

**Published:** 2023-08-18

**Authors:** Chenchen Liu, Qian Wu, Ranran Ren, Zhenyu Zhang, Yingjie Shi, Hongyun Li

**Affiliations:** a Department of Gastroenterology, Jining NO.1 People’s Hospital, Jining, Shandong Province, China; b Department of Ophthalmology, Affliated Hospital of Putian University, Putian, Fujian, China; c Department of Gastroenterology Nanjing First Hospital, Nanjing Medical University, Nanjing, Jiangsu, China; d Department of Infectious Diseases, Jining NO.1 People’s Hospital, Jining, Shandong Province, China.

**Keywords:** helicobacter pylori infection, meta-analysis, non-alcoholic fatty liver disease

## Abstract

**Background:**

The relationship between *Helicobacter pylori (H pylori*) infection and nonalcoholic fatty liver disease (NAFLD) has long been debated. Although it has been investigated in many observational studies, the results remain controversial. Therefore, we performed an updated meta-analysis to assess the association between *H pylori* infection and risk of NAFLD by collecting relevant articles.

**Methods:**

Literature collections were conducted by searching PubMed, EMBASE, Web of Science and Cochrane Library databases. Pooled odds ratios with corresponding 95% confidence intervals were calculated to estimate the strength of the link between *H pylori* infection and NAFLD using Stata 12.0 software.

**Results:**

28 studies with 68,047 cases of NAFLD patients and 134,866 controls were finally included in the meta-analysis. Overall, The results suggested a 27.5% increased risk of developing NAFLD in patients with *H pylori* infection (odds ratios 1.275 95% confidence intervals 1.179–1.379), although significant heterogeneity was observed. There is no significant publication bias observed based on the funnel plot and Begg test. Subgroup analysis revealed that variables of the study design, study region, publication year, and the method of diagnosing *H pylori* and NAFLD all contribute to the high heterogeneity, while the positive correlation was seen in all subgroup analysis.

**Conclusion:**

This meta-analysis disclosed 1.275-fold increased risk of the occurrence and development of NAFLD in *H pylori* (+) group compared with the *H pylori* (−) group, indicating that *H pylori* is a serious risk factor in patients susceptible to NAFLD.

## 1. Introduction

Nonalcoholic fatty liver disease (NAFLD) characterized by typical hepatic steatosis while excluding documented causes of liver diseases including alcohol use, is the most common chronic liver disease worldwide, and contributes to great clinical and financial burden globally.^[1]^The current prevalence of NAFLD worldwide is approximately 25%, varying across different regions, and the highest prevalence rates were observed in the Middle East (32%), followed by the region of South America (31%), 27.37% in Asia, 24.13% in North America, 23.71% in Europe.^[2–4]^ However, the exact etiology of NAFLD has not been fully elucidated, both environmental and metabolic factors may contribute to the underlying pathogenesis of NAFLD. Currently, there is still no efficient pharmacological therapy approved for NAFLD, under which condition patients of NAFLD might gradually progress toward liver fibrosis, cirrhosis, liver failure, and finally hepatocellular carcinoma. Thus, it is of vital importance to explore the etiology and pathogenesis of NAFLD.

*Helicobacter pylori (H pylori*), a well-known pathogen colonized in human stomach, first discovered by Marshall and Warren in 1982, is a gram-negative bacterium that increases the risk of developing chronic gastritis, gastric ulcer, gastric adenocarcinoma, and mucosa-associated lymphoid tissue lymphoma.^[5]^ *H pylori* infecting approximately 50% of the world population, has been paid attention to its extragastric manifestations currently. Many animal and clinical studies have suggested intestinal dysbiosis and *H pylori* infection may contributes to the occurrence and development of NAFLD.^[6–8]^ Unfortunately, the underlying mechanism of this phenomenon is still unclear. Recent studies have shown that *H pylori* infection may cause liver injury via some specific toxins,^[9]^ and the invasion of *H pylori* in the small bowel mucosa might increase gut permeability and thus facilitate the passage of bacterial endotoxins by the portal vein to the liver.^[10]^ The effect of *H pylori* infection on liver damage, has not been fully explored, and largerly remains unknown. Therefore, the early detection and diagnosis of NAFLD is of vital importance, and it is essential to identify possible risk factors for NFALD morbidity and progression.

Currently, established risk factors for NAFLD include insulin resistance, and some metabolic syndroms, namely, hypertension, obesity, dyslipidemia, type 2 diabetes mellitus. Recently, many studies have investigated the relationship between *H pylori* and NAFLD. Some concluded that *H pylori* infection is associated with an increased risk of NALFD while others yielded a null result. Hence, there is still insufficient evidence to implicate *H pylori* with NAFLD. Furthermore, several pertinent large observational studies have emerged in recent years. Therefore, we carried out an updated meta-analysis of all available published articles to evaluate the association between *H pylori* infection and the risk of NALFD.

## 2. Material and methods

### 2.1. Search strategy

We conducted a systemic search of the PubMed, EMBASE, Web of Science and Cochrane Library databases up to December 24th 2022 with the following search terms: (*H pylori* or *H pylori* or Hp or or *H pylori*) AND (nonalcoholic fatty liver disease or NAFLD or nonalcoholic steatohepatitis or NASH or nonalcoholic fatty liver or NAFL). The search was augmented by manual search of the reference lists of included articles for potentially relevant studies that might have been missed by the computer-assisted strategy. The literature search was stopped at December 24th, 2022.All analyses were based on previous published studies, thus no ethical approval and patient consent are required.

### 2.2. Selection criteria and quality assessment

We included studies that met the following inclusion criteria: Studies including patients with NAFLD; Study design: clinical trials including cohort studies, cross-sectional studies as well as case-control studies; Studies evaluating *H pylori* infection in patients; Sample sizes, odds ratios (ORs), and their 95% confidence intervals (CIs) and those with enough information for calculating these data; Full article in English language.

The exclusion criteria was as follows: Studies with other designs, including case reports, abstracts, laboratory and animal studies, reviews, and editorials; Studies that did not exclude individuals with significant alcohol intake and other documented causes of chronic liver disease; Non-English paper; Paper lacking ORs, and their 95% CIs and those with insufficient information for calculating these data; Studies performed in pediatric population. Full articles were obtained for studies that were potentially relevant. In addition, a recursive search of the reference lists of included studies was conducted to identify possible relevant articles by manual work. We assessed the quality of all included articles by the Newcastle–Ottawa quality assessment scale (NOS).^[11]^ Two authors (Chenchen Liu and Yingjie Shi) in the team assessed the methodological quality of each included study by the Newcastle–Ottawa quality assessment scale independently, which contains 9 items in total. The NOS Newcastle–Ottawa quality assessment scale assigns a maximum of 4 questions for selection, a maximum of 2 questions for comparability, and a maximum of 3 questions for exposure/outcome, with a maximum 1 point for each question. Points were scored only when the data were explicitly stated. Therefore, 9 points is the highest score, representing the highest quality of the studies. A third team member resolved any disagreements at this stage. The final decision and interpretation was based on consensus of 2 researchers (Chenchen Liu and Yingjie Shi) and when necessary with the assistance of Zhenyu Zhang. All selections and assessment were performed in duplicate.

### 2.3. Data extraction

Data were extracted independently by 2 members (Qian Wu and Ranran Ren). Any discrepancies in interpretation were resolved by consensus and when necessary with the assistance of the auhtor Hongyun Li. Relevant studies were reviewed in full article to ensure suitability according to the predefined inclusion and exclusion criteria. For each study included, we retrieved data by the list of the first author’s name, year of publication, country, study design, number of participants, the numbers of cases and controls, number of patients with NAFLD, number of patients with *H pylori* infection, diagnostic method of NAFLD and *H pylori*. We assessed the quality of all articles under the criteria of Newcastle–Ottawa. The final results were compared, and disagreements were discussed among all authors and were resolved with consensus. All data were crosschecked.

### 2.4. Statistical analysis

We used the Stata version 12.0 software (Stata Corporation, College Station, TX) to perform the meta-analyses. OR with 95% CI was used to describe the ratio of *H pylori* infection occurring in NAFLD patients versus the controls, which were retrieved from the published manuscripts or calculated from crude data in some studies if not available in the manuscript by this software. The pooled estimates were calculated by the inverse variance weighted estimation method. Heterogeneities of included articles were assessed based on Q statistics using the Mante-Haunszel weight and *I*^2^ statistics.^[12]^ Heterogeneity between studies was confirmed when studies yielded a *P* value of < .10 and an *I*^2^ value > 50 %. Heterogeneity was classified as follows based on the value of *I*^2^: a value of 0% to 25% indicating no heterogeneity, 26% to 50% low heterogeneity, 51% to 75% moderate heterogeneity, and 76% to 100% high heterogeneity. The fixed effect model was used when *I*^2^ values < 50%, and a random effect model was applied when *I*^2^ values > 50%. For studies with obvious heterogeneity, a random effects model was used based on the Der Simonian-Laird method. The forest plot was used to assess the relationship between *H pylori* infection and NAFLD. The potential publication bias was assessed graphically using Begg and Egger test and funnel plots. All *P* values were 2-sided, and *P* < .05 implicated statistical significance. To explore possible sources of heterogeneity, we conducted stratified analyses. To evaluate the sensitivity of the meta-analysis, we utilitied 1-way sensitivity analysis to evaluate the stability of our meta-analysis by sequentially excluding 1 study each time to test the robustness of our main results.

## 3. Results

### 3.1. Search results

**Figure 1.**
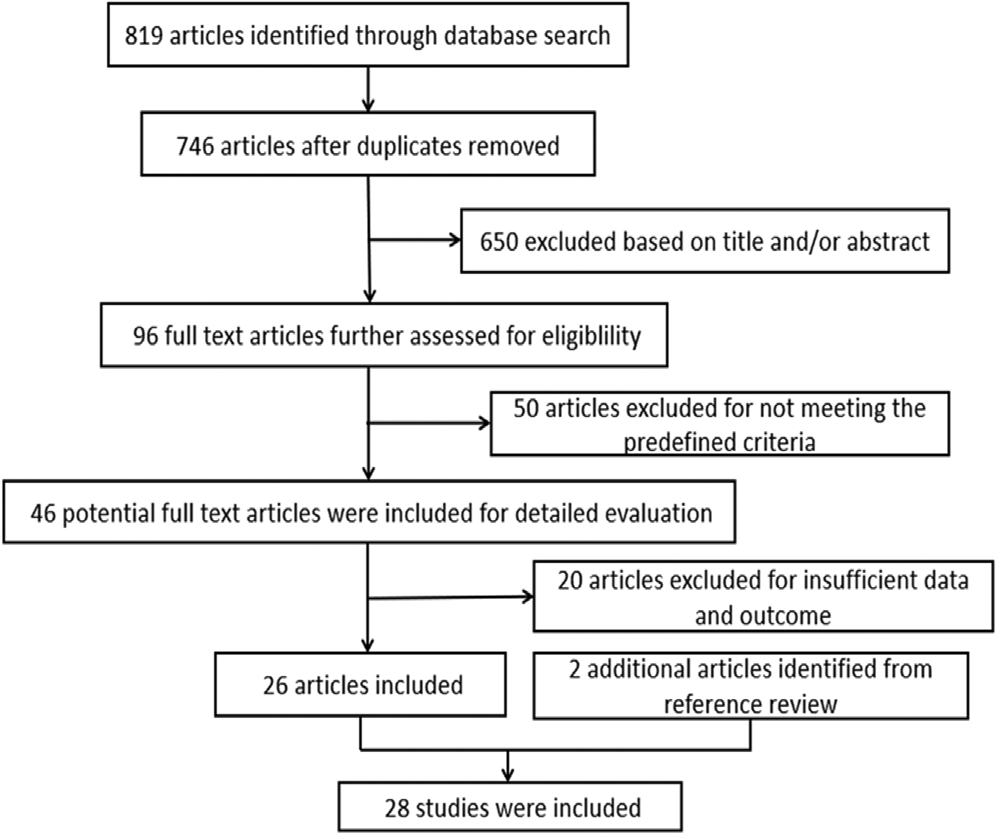
Study flow diagram.

By searching the aforementioned databases, we identified 819 citations, the titles and abstracts were then reviewed. After excluding duplicates, 73 irrelevant studies and duplicates were excluded, 746 citations were considered of potential value. Then, we further browsed the title, abstract, and full text of the literature, 650 citations were excluded based on the context. The full text of these 96 articles was further assessed for eligibility and retrieved for detailed evaluation. After further evaluation, 50 of them were subsequently excluded from the meta-analysis for not meeting the predefined criteria. Forty-six relevant paper were included for detailed evaluation, of which 20 articles were excluded for insufficient data and outcomes. Two additional articles were obtained by a manual search of different sources. Eventually, 28 articles published from April 2013 to February 2022 were included in the meta-analysis (Fig. 1). The related literatures included 2 cohort studies, 2 case-control studies, and 24 cross-sectional studies in our meta-analysis. The flow chart for the studies is shown in Figure 1.

### 3.2. Characteristics of included studies

A total of 203,313 participants were included, and the sample size for each study ranged from 53 to 71,633. Thirteen articles used the urea breath test, of which 10 studies used 13C-labeled urea breath test and 3 studies used 14C-labeled urea breath tests to confirm *H pylori* infection, 7 articles used serologic testing for antibodies to detect *H pylori* infection, stool antigen was used by 3 articles, and histologic test method was used by 3 articles, and 2 studies used multiply methods including urea breath test and serologic testing as well as the stool antigen method. Twenty-three articles used ultrasonography method to confirm the existence of NAFLD, and 3 articles used histologic method, 2 articles used other method including biopsy and the Hepatic steatosis index or NAFLD liver fat score. The study by Myong Ki Baeg et al^[13]^ provided 2 groups of data under 2 different criteria of NAFLD, namely the hepatic steatosis index and NAFLD liver fat score. Both groups of data met our inclusion criteria and were included in our meta-analysis. Among the 28 included studies, 12 studies were carried out in China,^[14–25]^ 3 in Egypt,^[7,26,27]^ 2 in Japan,^[28,29]^ 1 in Taiwan China,^[30]^ 1 studies were performed in the United States,^[31]^ 1 in Korea,^[13]^ 1 in Spain,^[32]^ 1 in Iran^[33]^ and 1 in Switzerland,^[34]^ 1 in Greece,^[35]^ 2 South Korea,^[36,37]^ 1 in Guatemala,^[38]^ 1 in Bangladesh^[39]^ (Table 1). In summary, 3 studies were performed in Africa and twenty in the Asia, and 5 studies were carried out in the western countries. The basic information about all included literatures were listed in Table 1.

**Table 1:** The characteristics of studies included in the meta-analysis.

First author	Year	Country	Study design	Hp diagnostic method	NAFLD diagnostic method	Total sample size	NAFLD group		Control group		Q/A score
							Hp+	Hp−	Hp+	Hp−	
S. A. Polyzos^[35]^	2013	Greece	Cross-sectional	Serology or UBT (C13)	Biopsy	53	23	5	14	11	6
T. J. Kim^[36]^	2017	South Korea	Cohort study	Serology	US	17,028	2080	1301	7838	5809	8
A. Abdel-Razik^[7]^	2018	Egypt	Cohort study	Stool antigen	US	369	12	0	159	198	8
S. J. Kang^[31]^	2018	USA	Cross-sectional	Serology	US	5404	658	1065	1115	2566	7
M. K. Baeg^[13]^	2016	Korea	Cross-sectional	UBT (C14)	Hepatic steatosis index or NAFLD liver fat score	3663	505	440	1131	1587	6
M. K. Baeg^[13]^	2016	Korea	Cross-sectional	UBT (C14)	Hepatic steatosis index or NAFLD liver fat score	3663	469	385	1167	1642	6
O. Cai^[14]^	2018	China	Cross-sectional	UBT (C13)	US	2051	145	288	500	1118	6
K. Okushin^[28]^	2015	Japan	Cross-sectional	Serology	US	5289	523	1279	926	2561	6
N. Fan^[15]^	2018	China	Cross-sectional	UBT (C14)	US	28,171	3905	5769	6943	11,554	7
Y. Y. Yu^[16]^	2018	China	Cross-sectional	UBT (C13)	US	20,389	3132	4460	4716	8081	7
T. Jiang^[17]^	2019	China	Cross-sectional	UBT (C13)	US	4081	1022	842	1115	1102	7
Y. E. E. Abo-Amer^[26]^	2020	Egypt	Cross-sectional	Stool antigen	US	646	442	82	96	26	7
M. Doulberis^[34]^	2020	Switzerland	Cross-sectional	Histologic	Histologic	64	15	0	40	9	6
C. S. Alvarez^[38]^	2020	Guatemala	Cross-sectional	Serology	US	424	222	29	145	28	7
M. Y. Xu^[18]^	2020	China	Cross-sectional	Serology	US	17,971	2516	2309	5287	7859	6
A. Lecube^[32]^	2016	Spain	Cross-sectional	Histologic	Histologic	416	264	110	25	17	6
C. Zhang^[19]^	2016	China	Case-control	UBT (C14)	Histologic	1200	300	300	144	456	8
C. X. Chen^[20]^	2017	China	Cross-sectional	UBT (C13)	US	2663	313	290	723	937	7
L. J. Lu^[21]^	2018	China	Cross-sectional	UBT (C13)	US	1867	199	397	390	881	7
L. Y. Yu^[30]^	2019	Taiwan	Cross-sectional	Histologic	US	2402	583	851	379	589	6
Y. Ping^[22]^	2021	China	Cross-sectional	UBT (C13)	US	1185	230	299	234	422	7
M. M. Rahman^[39]^	2020	Bangladesh	Cross-sectional	Serology	US	767	62	79	356	270	7
Y. M. Han^[29]^	2021	Japan	Cross-sectional	Serology	US	1784	343	528	365	548	7
Y. Liu^[23]^	2021	China	Cross-sectional	UBT (C13)	US	5665	1412	2543	685	1025	8
J. W. Wang^[24]^	2021	China	Cross-sectional	UBT (C13)	US	1898	199	306	490	903	7
W. J. Wang^[25]^	2022	China	Cross-sectional	UBT (C13)	US	71,633	8611	14,675	16,134	32,213	8
J. M. Choi^[37]^	2022	South Korea	Cross-sectional	UBT (C13)	US	2357	660	445	704	548	8
M. Mohammadifard^[33]^	2019	Iran	Cross-sectional	Serology, stool antigen	US	130	42	23	40	25	7
Abd El-Hamid E. Khalil^[27]^	2019	Egypt	Case-control	Stool antigen	US	80	42	18	6	14	7

US = ultrasonography.

### 3.3. Quality assessment

We assessed the quality of included articles by the Newcastle–Ottawa Quality Assessment checklists, and the results were as follows: 6 studies scored 8 points, 14 studies scored 7 points, and the other 8 studies scored 6 points. (Table 1)

### 3.4. Quantitative data synthesis

**Figure 2.**
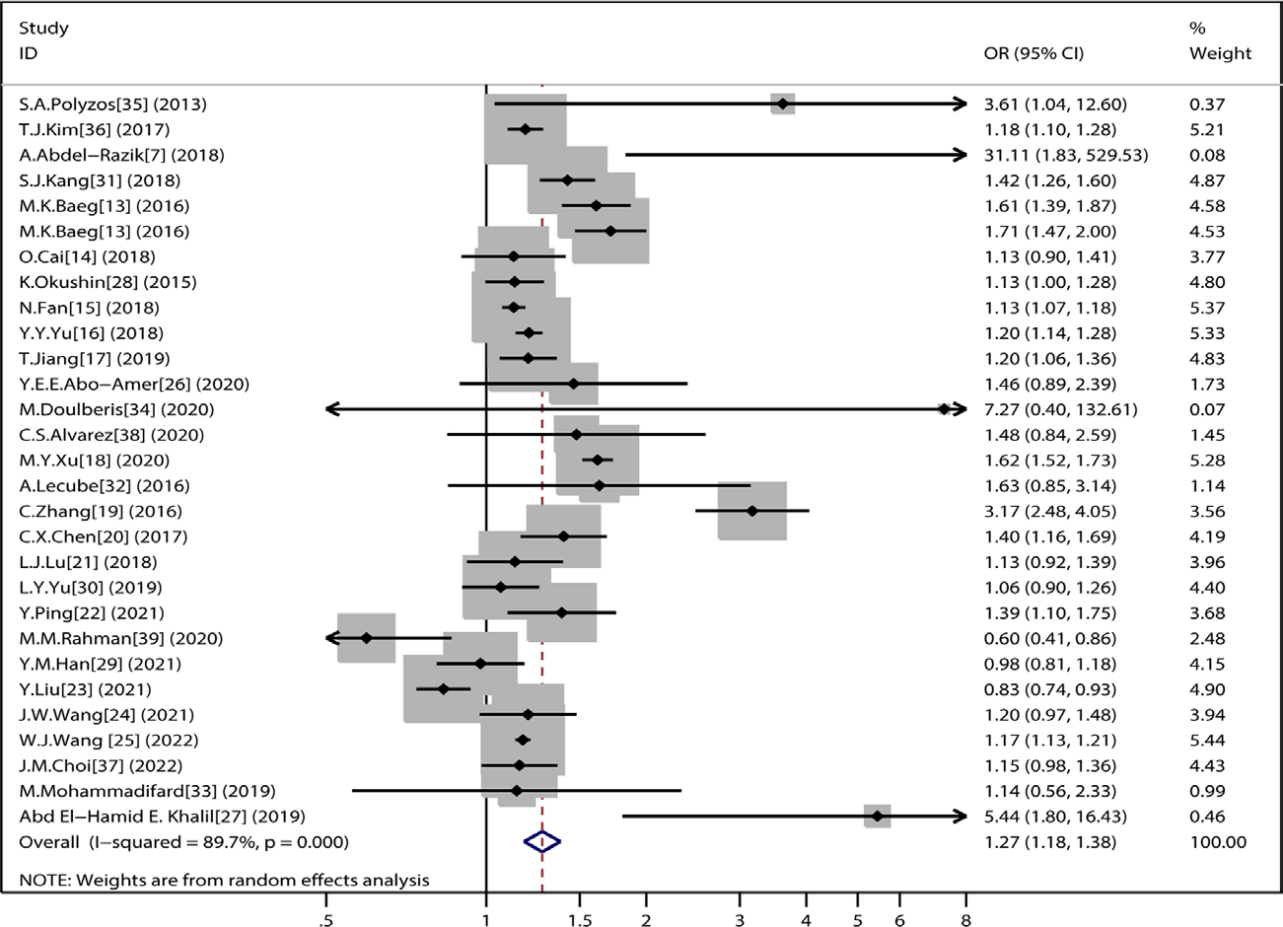
Random effects meta-analysis of studies evaluating *H pylori* infection and NAFLD.

These studies included a total of 68,047 cases of NAFLD patients and 134,866 controls. There were 83,096 *H pylori* (+) patients and 12,117 *H pylori* (−) patients in this meta-analysis. The OR ranged from 0.60 (95% CI 0.41–0.86) to 31.11 (95% CI 1.83–529.53). Most studies indicated an increase in the risk of NAFLD (OR > 1) in H.pyori patients. The pooled OR was 1.275 (95% CI 1.179–1.379; P heterogeneity = 0.000, *I*^2^ = 89.7%, Q = 270.64) (Fig. 2). The meta-analysis indicated that the odds of developing NAFLD was increased by 27.5% in *H pylori* (+) patients than in *H pylori* (−) patients (OR1.275 [95% CI 1.179–1.379]). The heterogeneity across studies was marginal (*P* = .00), therefore we performed subgroup analysis based on items of study design, the publication year, geographic character, *H pylori* diagnostic method, NAFLD diagnostic method.

### 3.5. Subgroup analysis

**Figure 3.**
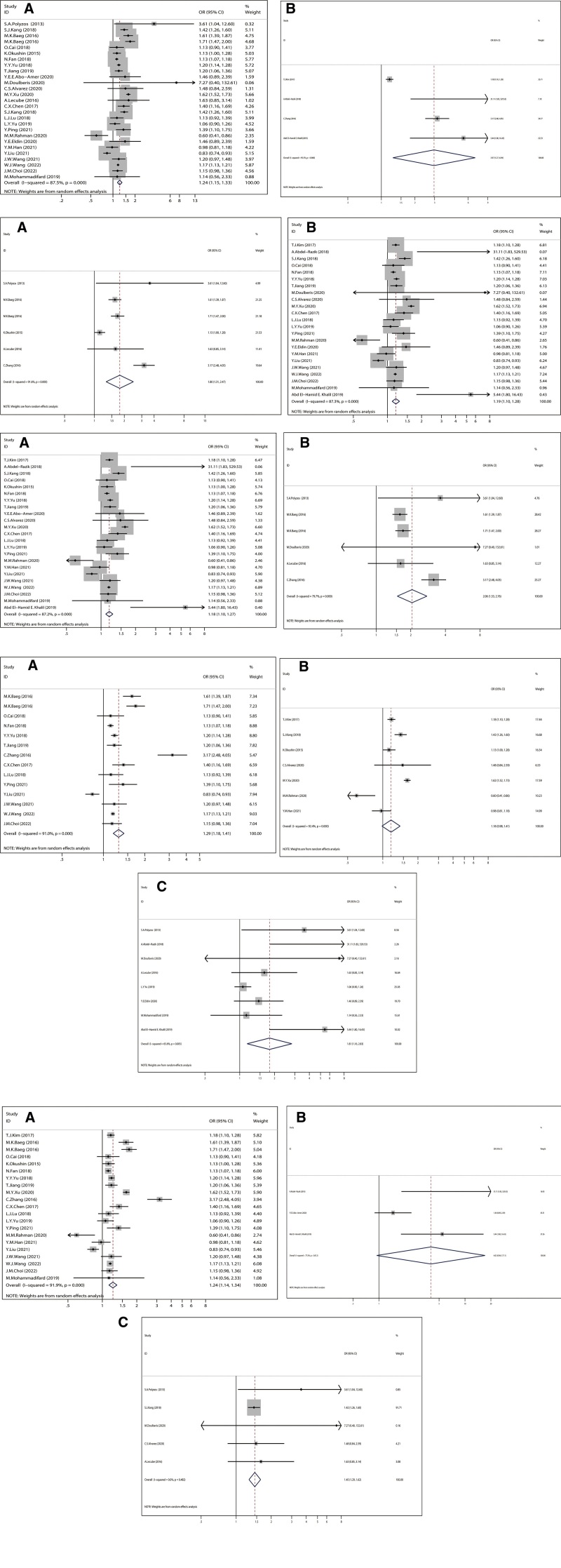
Stratified meta-analyses for *H pylori* infection and risk of NAFLD. (A). (A) Cross-sectional study, (B) the others. (B). (A) Asia group, (B) Africa group, (C) Western country group. (C). (A) Urea breath test (UBT) group, (B) serology group testing the antibody to *H pylori*, (C) the others. (D). (A) ultrasonography (US) group, (B) the others. (E). (A) 2016 and earlier, (B) those published after 2016.

In subgroup analysis based on items of study design, there were 24 cross-sectional studies, 2 cohort studies, 2 case-control studies in our meta-analysis. Since the numbers of cohort studies and case-control studies are too small to analysis separately, we conclude them together as the others. Similar to the results of the overall positive association, the pooled ORs for cross-sectional studies and the others were 1.235 (95% CI 1.149, 1.328, *I*^2^ 87.5%) and 2.965 (95% CI 1.267, 6.935, *I*^2^ 95.5%) respectively (Fig. 3A). In subgroup analysis by the criteria of geographical difference, the 28 studies included were divided into Africa group, Asia group and Western country group, of which the pooled ORs were 4.017 (95% CI 0.943, 17.105, *I*^2^ 77.5%, 1.237 (95% CI 1.140, 1.343, *I*^2^ 91.9%), 1.445 (95% CI 1.288, 1.621, 0.0%), respectively, all indicating a positive association between *H pylori* and NAFLD (Fig. 3B). Notably, the heterogeneity across studies among the Asia group was marginal, and that of the western country group was small. In subgroup analysis based on diagnostic method of *H pylori*, studies were divided into urea breath test group, serology group testing the antibody to *H pylori* and the others including stool antigen or histologic or multiple methods combining the above methods. The pooled ORs were 1.289 (95% CI 1.177,1.412, *I*^2^ 91.0%), 1.176 (95% CI 0.983,1.407, *I*^2^ 92.4%), 1.809 (95% CI 1.158,2.826, *I*^2^ 65.6%), respectively, all indicating a positive association (Fig. 3C). In subgroup analysis based on diagnostic method of NAFLD, studies were divided into ultrasonography group and the others including Histologic or Hepatic steatosis index or NAFLD liver fat score Hepatic steatosis index or NAFLD liver fat score, and the corresponding ORs were 1.181 (95% CI 1.100, 1.269, *I*^2^ 87.2%), 2.055 (95% CI 1.528, 2.764, *I*^2^ 79.7%), both indicating a positive association (Fig. 3D). When we perform the analysis by the criteria of publication year, studies were divided into the year of 2016 and earlier, and those published after 2016, the pooled ORs yielded were 1.801 (95% 1.312,2.472, *I*^2^ 91.6%), 1.186 (95%1.100,1.278, *I*^2^ 87.3%), respectively, both demonstrating positive association (Fig. 3E). According to the subgroup analysis, the study design, publication year, geographic character, *H pylori* diagnostic method, NAFLD diagnostic method were the factors that contributed to the high heterogeneity. The results of all subgroup analysis yielded a positive association between *H pylori* infection and NAFLD with different heterogeneity. Considering the limited number of studies included, we might take the conclusion critically and carefully. The results of all subgroup analyses were shown in Figure 3.

### 3.6. Publication bias

**Figure 4.**
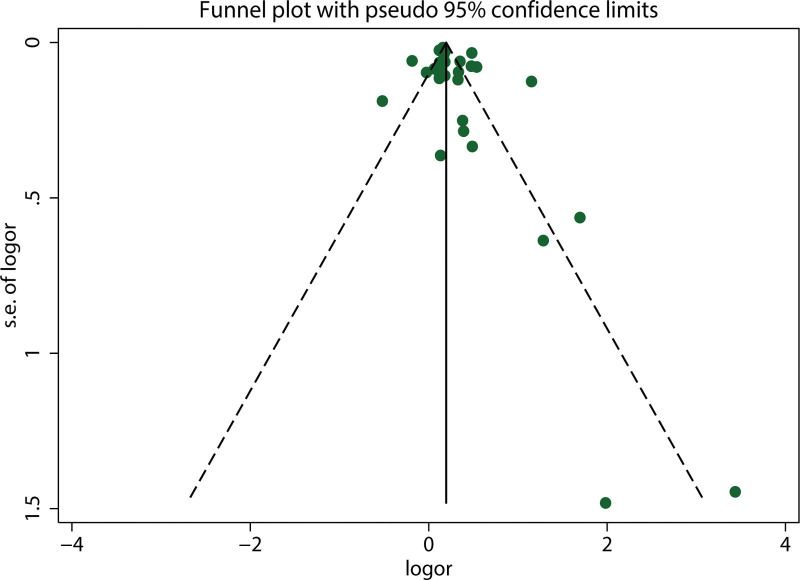
Funnel plot for the publication bias test of the included studies.

**Figure 5.**
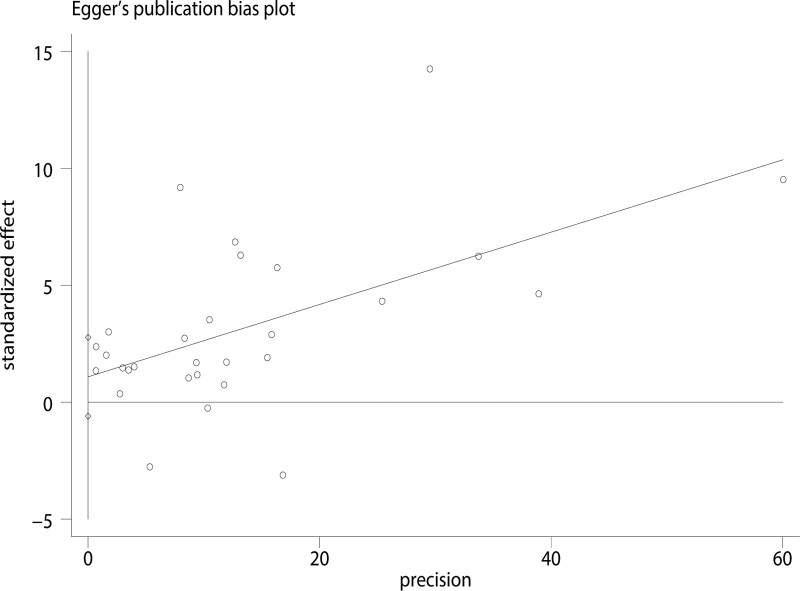
Publication bias of included studies using and Egger test.

Begg funnel plot and Egger test were performed to assess the publication bias of our meta-analysis. The shape of the funnel plots for included studies on the association between *H pylori* infection and the risk of NAFLD did not reveal any obvious asymmetry (Fig. 4). The *P* value for Egger linear regression method (*P* = .197) indicated that there was no statistical evidence of publication bias. Further, *P* value of Begg test was .063, which indicates no statistical publication bias (Fig. 5). Therefore, we assumed that there was no significant publication bias detected in our meta-analysis.

### 3.7. Sensitivity analysis

**Figure 6.**
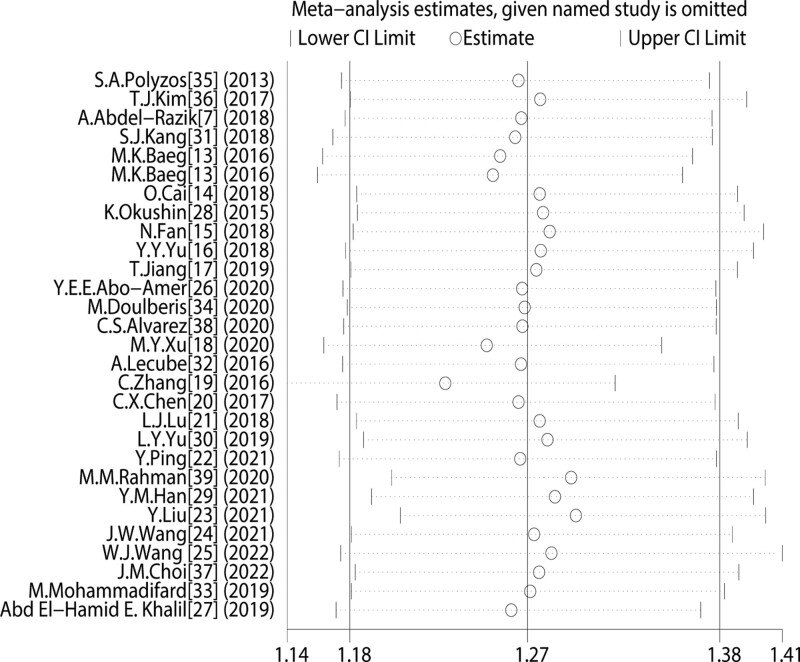
Sensitivity analysis of included studies.

To confirm the stability of the main results, we evaluated the influence of each single study on the results by systematically excluding 1 study each time and recalculating the overall summary estimates. Results show that no single study could markedly influence the significance of the summary ORs and the leave-1-out ORs ranged from 1.230 (95% CI 1.145, 1.322) to 1.300 (95% CI 1.206, 1.403), similar to the overall result. The statistical significance of the results was not altered when any single study was omitted, and thus confirmed the robustness of the results. (Fig. 6).

## 4. Discussion

The pathogenetic association between *H pylori* and NAFLD has long been in debate and clinical data were limited. The results of previous studies investigating association between *H pylori* and NAFLD have been inconsistent. Some systematic reviews showed that *H pylori* infection has a positive correlation with NAFLD, while some researchers reported a negative association. A large-scale cohort study conducted in Korea on 17,028 patients showed that *H pylori* infection was significantly associated with the development of NAFLD.^[36]^ Another investigation of 130 Japanese participants demonstrated that NAFLD is significantly higher in *H pylori*-infected patients in comparison with the noninfected controls.^[40]^ However, another study of 2051 participants concluded that *H pylori* infection did not appear to increase the risk of NAFLD.^[14]^ Baeg et al^[13]^ reported that *H pylori* infection did not increase the risk of NAFLD as indicated by hepatic steatosis index or NAFLD liver fat score. Additionally, no independent association between *H pylori* infection and NAFLD was found from a large population study conducted in the patients in China^[15]^ and Japan.^[28]^ And another Korean cross-sectional analysis of 3663 participants found that *H pylori* were not a possible cause of NAFLD too.^[13]^ Besides, some new relevant high-quality studies on the association between *H pylori* infection and NAFLD have emerged in recent years. Therefore, it is necessary to perform an updated meta-analysis on this issue to draw a more reliable conclusion.

Our meta-analysis disclosed 1.275-fold increased risk of progressing into NAFLD in *H pylori* (+) group compared with the *H pylori* (−) group, indicating that *H pylori* is a serious risk factor in patients susceptible to NAFLD. Our meta-analysis of updated published studies, using a detailed search strategy and selection criteria, provided convincing evidence that *H pylori* infection might be implicated in the progression of NAFLD. Our meta-analysis includes a larger sample size compared to previous studies and it is the most up to-date, which provided more reliable results.

As with any meta-analysis, our meta-analysis has some limitations. First, the majority of studies included were retrospective observational studies, making them susceptible to recall and selection bias. Second, significant heterogeneity was observed across studies, which might lower the reliability of the pooled OR estimates. Third, there exist some differences in the method of *H pylori* and NALFD diagnositic method; 4th, although we have included all the available studies in our meta-analysis, the number of studies and participants is still not enough. And therefore, the results should be taken and interpreted critically and carefully, and more multicenter prospective studies are necessary to confirm the main results of this meta-analysis.

## 5. Conclusion

We report a positive association between *H pylori* infection and an increased risk of NAFLD from an updated evidence. However, the precise role of *H pylori* in NAFLD still remains to be further explored considering the above limitations.

## Acknowledgements

The authors of the present study would like to thank Prof Ruijuan Liu (Department of Respiratory Medicine, Jining NO.1 People’s Hospital, China) for the help in writing and improving this paper.

## Author contributions

**Conceptualization:** Yingjie Shi.

**Data curation:** Qian Wu, Hongyun Li.

**Formal analysis:** Qian Wu.

**Investigation:** Chenchen Liu, Hongyun Li.

**Methodology:** Chenchen Liu, Ranran Ren, Zhenyu Zhang.

**Resources:** Zhenyu Zhang.

**Software:** Qian Wu, Ranran Ren, Yingjie Shi.

**Visualization:** Yingjie Shi.

**Writing – original draft:** Chenchen Liu, Qian Wu.

**Writing – review & editing:** Yingjie Shi, Hongyun Li.
